# Measuring Cot-Side the Effects of Parenteral Nutrition on Preterm Cortical Function

**DOI:** 10.3389/fnhum.2020.00069

**Published:** 2020-03-17

**Authors:** Viviana Marchi, Nathan Stevenson, Ninah Koolen, Raffaele Mazziotti, Francesca Moscuzza, Stefano Salvadori, Rossella Pieri, Paolo Ghirri, Andrea Guzzetta, Sampsa Vanhatalo

**Affiliations:** ^1^Institute of Life Sciences, Scuola Superiore San’Anna, Pisa, Italy; ^2^Department of Developmental Neuroscience, IRCCS Fondazione Stella Maris, Pisa, Italy; ^3^BABA Center, Pediatric Research Center, Children’s Hospital, Helsinki University Hospital, Helsinki, Finland; ^4^Department of Clinical Neurophysiology and Neuroscience Center, University of Helsinki and Helsinki University Hospital, Helsinki, Finland; ^5^Brain Modelling Group, QIMR Berghofer Medical Research Institute, Brisbane, QLD, Australia; ^6^Institute of Neuroscience, National Research Council, Pisa, Italy; ^7^Department of Maternal and Child Health, Division of Neonatology and Neonatal Intensive Care Unit, Santa Chiara Hospital, University of Pisa, Pisa, Italy; ^8^Institute of Clinical Physiology, National Research Council, Pisa, Italy; ^9^Department of Clinical and Experimental Medicine, University of Pisa, Pisa, Italy

**Keywords:** preterm nutrition, functional biomarker, neonatal EEG, preterm infant, neonatal intensive care unit, brain monitoring, computational EEG analysis

## Abstract

Early nutritional compromise after preterm birth is shown to affect long-term neurodevelopment, however, there has been a lack of early functional measures of nutritional effects. Recent progress in computational electroencephalography (EEG) analysis has provided means to measure the early maturation of cortical activity. Our study aimed to explore whether computational metrics of early sequential EEG recordings could reflect early nutritional care measured by energy and macronutrient intake in the first week of life. A higher energy or macronutrient intake was assumed to associate with improved development of the cortical activity. We analyzed multichannel EEG recorded at 32 weeks (32.4 ± 0.7) and 36 weeks (36.6 ± 0.9) of postmenstrual age in a cohort of 28 preterm infants born before 32 weeks of postmenstrual age (range: 24.3–32 weeks). We computed several quantitative EEG measures from epochs of quiet sleep (QS): (i) spectral power; (ii) continuity; (iii) interhemispheric synchrony, as well as (iv) the recently developed estimate of maturational age. Parenteral nutritional intake from day 1 to day 7 was monitored and clinical factors collected. Lower calories and carbohydrates were found to correlate with a higher reduction of spectral amplitude in the delta band. Lower protein amount associated with higher discontinuity. Both higher proteins and lipids intake correlated with a more developmental increase in interhemispheric synchrony as well as with better progress in the estimate of EEG maturational age (EMA). Our study shows that early nutritional balance after preterm birth may influence subsequent maturation of brain activity in a way that can be observed with several intuitively reasoned and transparent computational EEG metrics. Such measures could become early functional biomarkers that hold promise for benchmarking in the future development of therapeutic interventions.

## Introduction

The increasing survival of extremely preterm birth has brought attention to improving care practices for better long term outcomes (Ancel et al., 2015). Early nutrition is a key component of successful care in the neonatal intensive care unit (NICU; Stephens et al., 2009; Strømmen et al., 2015), and its compromise implies significant physiological stress (Can et al., 2012; Pampanini et al., 2015) with effects on structural brain maturation as well as long-term neurodevelopment (Stephens et al., 2009; Keunen et al., 2015).

Developing better nutritional paradigms has been challenged by the need to use the long-term neurodevelopmental outcome as the therapeutic endpoint. Long-term outcomes, however, imply very long study cycles and are confounded by a myriad of factors facing the infant after discharge from the NICU (Keunen et al., 2015). Outcome measures that are more proximal allow the assessment of therapeutic efficacy within days or weeks of the treatment. Advanced brain imaging was recently reported to yield measures of the brain’s structural development that correlate with the early parenteral and enteral nutrition in preterm infants (Vinall et al., 2013; Coviello et al., 2018; Schneider et al., 2018; Cormack et al., 2019). Magnetic Resonance Imaging (MRI) is, however, not routinely available for cot-side tracking of the brain’s development and requires substantial resources.

Scalp-recorded electroencephalography (EEG) is a non-invasive and readily available cot-side measure of cortical function. It is also extensively used in the NICU for long-term brain monitoring. Early studies using neonatal EEG have shown that poor nutritional status may affect the maturation of visually assessed EEG activity and is associated with delayed development (Hayakawa et al., 2003; Okumura et al., 2010), and more recently amplitude-integrated EEG was used to report effects of nutrition on early brain maturation in extremely preterms (Binder et al., 2019). While these results are based on visual EEG review of infants with exceptional nutritional deficiencies, they suggest that the EEG may provide a developmentally valid, functional measure of the preterm infant brain. Several novel computational metrics have been developed to better assess brain maturation. They are commonly based on the assumption that postmenstrual age can be used as a contextual benchmark to permit an objective assessment of cortical activity. These methods have the potential to track cortical maturation over preterm development (Koolen et al., 2016; Stevenson et al., 2017) by quantifying changes in spectral power (Niemarkt et al., 2011; Jennekens et al., 2012), increases in continuity [i.e., reduction in discontinuity, or interburst intervals (IBIs; Niemarkt et al., 2010; Koolen et al., 2014; Videman et al., 2016)], and increases in hemispheric synchrony [activation synchrony index; ASI (Koolen et al., 2016)]. A combination of these and other computational features using machine learning paradigms allows automated estimation of EEG maturational age (EMA; Stevenson et al., 2017). The EMA can be considered a global maturational index for serial recordings in preterm infants, which makes it highly applicable to the development of proximal outcome markers in studies on therapeutic interventions, such as parenteral nutrition.

Here, we set out to study whether computational measures of preterm EEG could be used to detect differences in functional brain maturation related to early nutritional status in the early preterm infants. We tested the hypothesis that changes in computational metrics of serial EEG correlates with early parenteral nutrition and also with the long term clinical outcome.

## Materials and Methods

### Patients

We recruited preterm infants born at ≤32 weeks of gestation with birth weight ≤1,500 g (i.e., very or extremely low birth weight infants), born at the Pisa University Hospital, Italy. The study initially included a clinical observational study with a nutrition protocol as well as two consecutive EEG recordings planned at 32 and 36 weeks post-menstrual age (PMA). The infants attended the outpatient neurological and neonatological follow up at the NICU of the Pisa University Hospital until they reached 24 months of corrected age by June 2017. The parents gave written consent, the use of the data in scientific research was approved by the Azienda Ospedaliera—University Hospital of Pisa.

We excluded newborns with suspected genetic abnormalities, inborn errors of metabolism, or major malformations connected to postnatal growth defects and those who developed brain parenchymal lesion (clear cystic periventricular leukomalacia (cPVL) and/or intraventricular hemorrhage (IVH) grade III or higher (defined using the classification of Papile et al., 1978). Infants performing examinations outside the reference time frame, with poor quality EEG recordings or not attending the follow-up clinic, were also excluded.

### Clinical Data

#### Clinical Data

Weight, head circumference (HC) and length were measured at birth and converted to z-scores based on Italian population growth charts (Bertino et al., 2010). We recorded newborns as small for gestational age (SGA) if their weight was less than the 10th percentile for PMA at birth.

Variables related to clinical complications of prematurity were reported including mechanical ventilation duration, bronchopulmonary dysplasia (defined as ventilatory or oxygen supply at 36 weeks’ PMA), and sepsis (defined as culture-proven infection or as clinical signs with inflammatory syndrome (C-reactive protein >20 mg/l) leading to greater than or equal to 7 days of antibiotic treatment). Necrotizing enterocolitis (NEC) was defined using Bell’s classification as stage II or more. Patent ductus arteriosus (PDA), diagnosed on echocardiography was considered clinically significant when it required medical treatment. Each infant had a cranial ultrasound every 2 weeks according to routine protocols.

#### Nutritional Data

Parenteral and enteral nutrition were monitored daily from birth until the end of parenteral support. Nutritional data were collected on the duration of parenteral nutrition, time of initiating enteral feeding and full enteral feeding time. The total daily intake was calculated by averaging enteral and parenteral contributions (expressed as ml/kg/day) and composition of macronutrients, specifying the mean daily value in the first 7 days of life (1–7 day of life; DoL) for proteins (amino acids; g/kg/day), lipids (g/kg/day) and carbohydrates (g/kg/day). Mean daily energy intake (kcal/kg/day) was computed with proteins and carbohydrates providing 4 kilocalories (kcal) per gram and lipids providing 9 kcal per gram.

#### Nutritional Protocols

The nutrition protocols included starting parenteral nutrition upon admission to the NICU, as well as encouraging breastfeeding; early trophic feeds were used as minimal enteral nutrition, with increasing dosage, until the shift to total enteral nutrition. Intake of fluid, glucose and electrolytes were ordered by the attending neonatologist and were recorded, parallel to parenteral and enteral values. The nutritional protocol changed slightly in the recent period, as since January 2014, it was standardized according to the national guidelines, published by Italian Society of Neonatology in 2013 (Romagnoli, 2018) based on the instructions published by ESPHAGAN/ESPEN/ESPR in 2005 (Koletzko et al., 2005) and thus giving opportunity to include a wide spectrum of nutritional statuses in the cohort. The standardized nutritional protocol started in 2014, implying that infants would start parenteral nutrition in the first day of life with an intake of 2.5 g/kg per day amino acids and 1.5 g/kg per day lipids, followed by a gradual increment for a target intake of 4.0 g/kg/day amino acids and 3.0 g/kg per day lipids. Glucose intake was calculated to start with 6 g/kg/day, and progressively increased to a target of 15 g/kg/day at the tenth day of life; parenteral amino acid dosage was reduced when enteral feeding supplied 0.5 g/kg/day protein and was terminated when enteral feeding supplied 75% of total nutrition volume.

#### Outcome Data

Neurodevelopmental outcome was assessed using clinical referrals collected during a follow-up program with neonatological and neurological visits. An experienced neurologist (author AG) determined clinical outcomes by retrospective review of the medical records using the predefined outcome categories (see also Iyer et al., 2014): normal, mild and moderate abnormality (including mild speech, motor, or cognitive delay, autism and cerebral palsy (CP) without cognitive problems), or severe abnormality (including tetraplegic and/or severe dyskinetic CP, severe mental retardation, and severe epilepsy).

### EEG Recordings

All subjects underwent two consecutive EEG examinations during their stay in NICU. The first EEG was performed at about 32 weeks PMA (T1; mean: 32.4 weeks PMA, range: 30.4–33.6 at 27 days after birth, range: 6–54), and the second EEG was performed at about 36 weeks PMA (T2; mean: 36.7 weeks, range: 35.3–39.1), so the interval between recordings varied between 3 and 7 weeks, with a mean of 4.3 weeks. All infants were clinically stable at the time of EEG recording.

**Figure 1:**
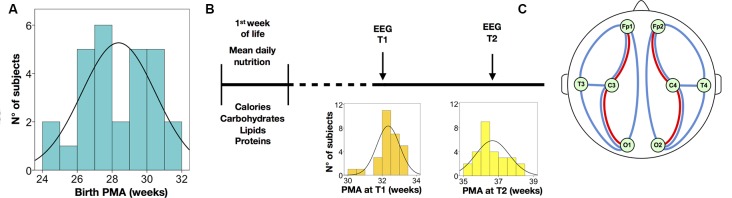
Summary of the study protocol and electroencephalography (EEG) analysis. **(A)** Distribution of postmenstrual age at birth. **(B)** Timeline of nutritional data and EEG assessments; the distribution of post-menstrual age (PMA) is displayed at the EEG at T1 and T2, respectively. **(C)** Schematic drawing of the electrode placement used in the study; the bipolar derivations depict signals used for quantifying interburst interval (IBI; red lines) and activation synchrony index (ASI; blue lines); spectral analyses were calculated from all electrodes; ASI and IBI were also estimated from all electrode pairs.

Multi-channel EEG recordings were acquired using a Brain Quick/ICU EEG (MicroMed, Treviso, Italy) with a referential montage from 9 scalp electrodes located at Fp1, Fp2, C3, C4, O1, O2, T3, T4 (international 10–20 system), plus a nasal reference electrode, applied to the scalp using adhesive paste. Unless otherwise stated, the visual analyses were performed on a standard bipolar montage: Fp1-C3, C3-O1, Fp1-T3, T3-O1, Fp2-C4, C4-O2, Fp2-T4, T4-O2 (see Figure 1). A low-pass filter with a cutoff of 70 Hz and a high-pass filter with a cut-off of 0.3 Hz was applied. The EEG recordings were sampled at 256 Hz.

The EEG recordings lasted at least 40 min and included at least one full sleep-wake cycle, including both active and quiet sleep (QS). The sleep states were defined by careful visual inspection of the bipolar montage by one of the authors with extensive experience in neonatal EEG (VM); blind to the clinical information of the given infant. All EEGs were visually reviewed for medical diagnostics.

### EEG Analysis

Quantitative EEG assessment consisted of four well-known features of neonatal EEG: (1) spectral power during QS; (2) IBIs during QS as well as the global level of discontinuity expressed as Mean Suppression Curve (MSC); (3) interhemispheric synchrony (ASI) during QS; and (4) an EMA estimate.

The full Matlab scripts for IBI (Jennekens et al., 2012; Videman et al., 2016), MSC (Niemarkt et al., 2011; Koolen et al., 2014), ASI (Räsänen et al., 2013; Romagnoli, 2018) and EMA (Koolen et al., 2016; Stevenson et al., 2017) have been published previously. Spectral analysis, IBI, MSC, and ASI were run on visually selected periods of QS, while EEG data for the calculation of EMA was run on the whole recording. Many of the hereby used algorithms can be found in an open repository (link GitHub: https://github.com/nstevensonUH/Neonatal-EEG-Analysis.git and https://github.com/vivi-mar/spectralEEG_preterm).

QS epochs with sufficient signal quality were obtained from 29 (93%) at T1 and from 27 (90%) at T2, with EEG epochs from both T1 and T2 available in 25 (80%) of patients. A minimum of 5 min of continuous EEG was required for MSC, IBI, and ASI, hence they were available for analysis from 26 EEG recordings (87%) at T1 and from 25 EEG recordings (83%) at T2.

#### Spectral Analysis

The power spectral densities (PSD) were computed from the eight referential derivations (Fp1, Fp2, C3, C4, T3, T4, O1, O2) in each epoch of QS. We used Welch’s method to estimate the PSD (2 s epochs with 50% overlap; Hamming window), calculated as the integral of EEG power for each frequency band: Delta1, 0.5–1.0 Hz; Delta2, 1.0–4 Hz; Theta, 4.0–8.0 Hz; Alpha, 8.0–13.0 Hz; and Beta, 13.0–30.0 Hz and according to Niemarkt et al. (2011) and Jennekens et al. (2012). We also accounted for the total bands EEG powers, 0.5–30.0 Hz, that was expressed as global spectra. For each frequency band, we calculated: (1) absolute spectra, defined as the integral of EEG powers within the frequency band, that we expressed in μV^2^ (abbreviated to Abs); and (2) relative power, defined by the ratio of every absolute band power to the total bands power and expressed in percentage (abbreviated to Rel). The spectral algorithm can be found in the open repository (see the Github link: https://github.com/vivi-mar/spectralEEG_preterm).

PSD was calculated from all eight channels (total) and also averaged across the following signals: frontal (Fp1 and Fp2), central (C3 and C4), occipital (O1 and O2) and centrotemporal right (C4 and T4) and left (C3 and T3).

#### Continuity

The level of continuity was evaluated both globally and on brain regions, using two measures, the MSC as well as the 95th percentile of IBI intervals (IBI 95) using a previously published method of automated burst detection (Koolen et al., 2014). Average IBI duration was also calculated on Centro-occipital (C3-O1 and C4-O2) and fronto-central derivations (Fp1-C3, Fp2-C4; see also Jennekens et al., 2012). The measure of IBI in fronto-central derivations was estimated from 26 EEG recordings at T1, and from 21 EEG recordings at T2.

#### Synchrony of Cortical Activations

The interhemispheric synchrony was estimated using a recently developed measure, activation synchrony index (ASI; Romagnoli, 2018; Binder et al., 2019), which estimates the statistical co-occurrence of activations, or intermittent rise in EEG activity, that characterizes the background EEG during QS in newborn infants. The analysis parameters, such as filtering and window lengths of ASI were optimized to emulate the visual assessment of interhemispheric synchrony that is a standard component of visual EEG review in the clinical interpretation and known to correlate with neurological compromise in a large array of clinical conditions (Räsänen et al., 2013). ASI analysis was calculated in two ways (Binder et al., 2019). First as the average of all 28 pairwise ASI values to characterize global connectivity. Second, as the average of symmetric channel combinations to assess interhemispheric synchrony, and between symmetric pairs of bipolar derivations: fronto-central (Fp-C), fronto-temporal (Fp-T), fronto-occipital (Fp-O), Centro-occipital (C-O), temporo-occipital (T-O) and centrotemporal (C-T).

#### EEG Maturational Age (EMA)

Automated estimate of EMA provides a global estimate of EEG maturation in weeks. The EMA algorithm is described in full detail in the original publication (Stevenson et al., 2017) and can be found in the open repository (see the Github link: https://github.com/nstevensonUH/Neonatal-EEG-Analysis.git).

The EMA is a machine learning-based computational measure that uses a large set of heuristic features that were used originally to train the algorithm using a cohort of healthy preterm infants from another center (Vienna, see Koolen et al., 2016). In the original implementation, EMA was trained to use several hours of unselected EEG data, however, our present dataset was more restricted with respect to data lengths. Due to slightly varying recording methods and many other such clinical factors, there was an expected offset between EMAs and the actual age of the infants in this study. We used the change in this offset (EMA gap; the distance of EMA from the actual age) to measure maturation; the EMA gap was compared to the difference in PMA between T1 and T2 to assess whether an infant’s development is delayed or advanced.

### Statistical Analysis

Results were statistically analyzed using the Statistical Package for the Social Sciences (SPSS, version 23.0; IBM SPSS, Chicago, IL, USA, RRID:SCR_002865). As in the recent study of Schneider et al. (2018), we used Generalized Estimating Equation (GEE) models accounting for repeated measures, to test the effect of nutritional variable and age on each maturational feature. The GEE model was run with an independent correlation structure and robust Standard Error (SE) adjustment for age at the EEG recording. We built separate models for each macronutrient (expressed as the mean dose in g per kg per day). Group differences between outcome categories were studied using a non-parametric test (Mann–Whitney U test). The statistical significance was set as *p* < 0.05.

## Results

### Demographic and Clinical Characteristics of the Sample

Out of an initial cohort of 60 infants monitored with early nutritional analysis, serial EEG recordings were available in 36 infants (60%), and in the remaining 24 only one EEG was available. Eight of these were then excluded from the study: one had a diagnosis of biotinidase deficiency; three had EEG exams only after term age and one of those had also bad quality EEG recordings, two developed PVL with cystic pattern at cranial US and two were lost to follow-up. A total of 28 (46% of the total dataset) infants were included in the study; clinical, demographic characteristics and reports of perinatal clinical events are shown in Table 1.

**Table 1:** Study population.

	Mean ± SD	min-max
PMA at birth (weeks)	28.4 ± 2.1	24.3–32.0
Birth weight (kg)	1,022.4 ± 204.3	637.0–1352.0
Birth length (cm)	34.88 ± 2.39	29.00–39.00
Birth head circ. (cm)	25.79 ± 2.12	21.50–29.00
Day of discharge	78.1 ± 29.3	38.0–161.0
PMA discharge (weeks)	39.6 ± 2.8	36.3–47.3
PMA at T1(weeks)	32.4 ± 0.7	30.4–33.6
PMA at T2 (weeks)	36.7 ± 0.9	35.3–39.1
Time T1-T2 (days)	30 ± 8	21–49

*Legend: PMA, post menstrual age; wks, weeks; SD, standard deviation; min-max, range of values*.

All infants were fed from day one with parenteral nutrition, which was discontinued after a mean of 24 days (ranging from 10–58 days). In the first week of life, the mean calories administered were 70.01 ± 6.29 kcal/kg/day, globally accounting for the administered quantities in the different macronutrients, respectively: carbohydrates (mean 10.31 ± 0.72 g/kg/day), lipids (mean 2.03 ± 0.42 g/kg/day) and proteins (2.54 ± 0.36 g/kg/day).

Four infants (14%) presented with patent arterial ductus, three (11%) had sepsis while none of them showed signs of NEC. Bronchopulmonary Dysplasia (BPD) was present in seven infants (75%).

Based on follow up clinical reports, 16 infants (57%) had normal development, 12 (43%) presented with mild abnormalities, such as developmental delays or minor neurological abnormalities; only one presented with mild diplegic CP which was classified as minor abnormality in this study. Notably, the infants with normal development were younger at birth (PMA at birth was 27.5 vs. 29.4 weeks; *p* = 0.033, *n* = 16) and they received more protein during the first week of life (mean daily amount of 2.67 vs. 2.37 g/kg/day; *p* = 0.007, *n* = 12).

### Nutrition Effects on EEG Spectra

**Figure 2:**
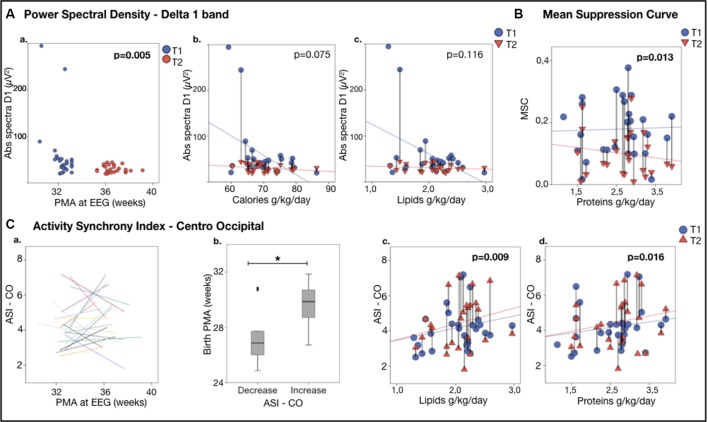
EEG featuresvs. nutrition. **(Aa)** Power Spectral Density for Delta 1 band: Delta 1 band power (absolute spectra) vs. age at the recording.**(Ab,c)** Delta 1 band power vs. nutrient intake during the first week of life. Note the significant decline in the delta 1 poweras a function of calories **(Ab)** and lipids **(Ac)**. **(B)** Continuity, expressed as Mean Suppression Curve (MSC) vs. nutrition; note the significant higher increase from T1 to T2 in MSC as a function of the mean daily protein intake in the first week of life. **(C)** Activity Synchrony Index over Centro-Occipital areas vs. nutrition. **(Ca)** Individual trajectories of Centro-Occipital ASI (C-O ASI) as a function of age, showing that in half of the individuals the ASI increased. **(Cb)** A comparison of these subgroups showed that gestational age at birth was higher in the infants, showing ASI increase (29.6 ± 1.6 vs. 27.7 ± 2.3 weeks PMA, *p = 0.023). C-O ASI vs. 1-week lipids **(Cc)** and proteins **(Cd)** intake. Vertical lines connect individuals’ EEG metrics from T1 and T2; *p*-values refer to generalized estimating equation (GEE) models that account for PMA at both recordings.

The EEG spectra showed an overall developmental change between the two recordings, by showing a reduction in both absolute and relative spectral powers in the Delta 1 band along the increasing age (Abs Delta 1: *B* = −7.186, *p* < 0.011 (see Figure 2Aa and Table 2); Rel Delta 1: *B* = −2.307, *p* = 0.000; *n* = 28) while there was an increase in relative spectral power in the Delta 2 band, theta and alpha band (Rel Delta 2: *B* = 1.407, *p* = 0.000; Rel Theta: *B* = 0.266, *p* = 0.004; Rel Alpha: *B* = 0.108, *p* = 0.002). Regional comparisons showed that the developmental effects were seen over frontal, central, and centrotemporal areas, being in line with the previous studies on the spectral maturation of brain activity (Niemarkt et al., 2011; Jennekens et al., 2012).

**Table 2:** Generalized Estimation Equation model (GEE) to study associations between nutritional intake (mean daily intake) in the 1-week of life as a predictor for the changes in EEG measures.

Mean daily nutritional supply-1–7 DoL	Abs Delta 1		MSC		CO ASI		EMA gap	
Calories (kcal/kg/day)	*B* = −2.374	*p* = 0.075	*B* = 0.011	*p* = 0.171	*B* = 0.042	*p* = 0.110	*B* = −0.024	*p* = 0.220
Carbohydrates (g/kg/day)	*B* = −5.867	*p* = 0.140	*B* = −0.031	*p* = 0.510	*B* = −0.291	*p* = 0.275	*B* = −0.194	*p* = 0.288
Lipids (g/kg/day)	*B* = −34.767	*p* = 0.116	*B* = 0.153	*p* = 0.274	*B* = −0.884	*p* = 0.009	*B* = 0.595	*p* = 0.042
Proteins (g/kg/day)	*B* = −0.788	*p* = 0.441	*B* = 0.419	*p* = 0.013	*B* = 1.172	*p* = 0.016	*B* = 1.108	*p* = 0.000
Post menstrual age (weeks)	*B* = −7.186	*p* = 0.011	*B* = −0.023	*p* = 0.006	*B* =0.032	*p* = 0.609		

*MSC, mean suppression curve; IBI, Centro-Occipital Interrupts Interval; ASI, Activity Synchrony Index; CO, Centro-Occipital; EMA gap, difference in weeks between estimated maturational age and postmenstrual age*.

Higher nutritional intake trended with greater reduction in the absolute amplitudes in the delta 1 band as well as in the global spectra, as expected by typical development (Abs Delta 1: calories: *B* = −2.374, *p* = 0.075, lipids: *B* = −34.767, *p* = 0.116; see Figures 2Bb,c; Abs Tot: calories *B* = −3.555, *p* = 0.098 *n* = 28, results adjusted for age).

Spatial comparisons suggested that the effects of nutrient intake to spectral changes are global, with the most prominent effects in absolute spectral power in the Delta 1 band over the right centrotemporal areas (calories: *B* = −4.073, *p* = 0.066; *n* = 28).

### Nutrition Effects on Continuity

A significant developmental change was found in the global measures of continuity. Both the maximal IBI (IBI 95: *B* = −0.849, *p* < 0.001, *n* = 26) and MSC (*B* = −0.025; *p* = 0.001, *n* = 28) decreased with age, being in line with the previous works on continuity (Koolen et al., 2014; Videman et al., 2016).

Higher proteins in the early days of life were slightly associated with a greater reduction in MSC (*B* = 0.419, *p* = 0.013, *n* = 28; see Figure 2B).

### Nutrition Effects on Cortical Synchrony

ASI was decreased during maturation in both fronto-temporal (F-T ASI: *B* = −0.158, *p* = 0.002, *n* = 25, for three of them there was no enough sleep in both the consecutive recordings) and centrotemporal derivations (C-T ASI: *B* = −0.131, *p* = 0.002, *n* = 25).

The effects of early nutrition were stronger over posterior regions. A higher amount of protein or lipid intake in the early days of life was associated with more advanced maturation reflected by a higher increase in ASI in the Centro-occipital derivation (C-O ASI—lipids: *B* = 0.884, *p* = 0.009; proteins: *B* = 1.172 *p* = 0.016, *n* = 25; see Figures 2Cc,d).

Closer inspection of individual ASI trajectories (Koolen et al., 2016) suggested that there may be two groups with different developmental changes in C-O ASI by comparing the two recordings: some individuals showed a clear increase in ASI while others showed a decrease; Figures 2Ca,b). Infants with increased ASI had a significantly higher GA compared to those who presented a decrease (*p* = 0.023; 29.6 ± 1.6 weeks, *n* = 12 vs. 27.7 ± 2.3 weeks *n* = 13 of GA).

### Nutrition Effects on Estimated Maturational Age

**Figure 3:**
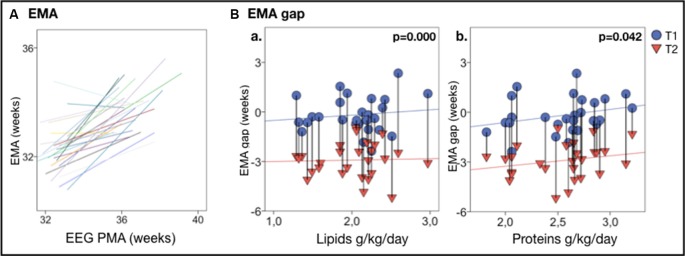
EEG maturational age (EMA) gap vs. nutrition. **(A)** Individual trajectories of EMA are highly correlated with the actual PMA; a clear developmental increase in EMA between T1 and T2 is displayed in nearly all infants. **(B)** EMA gap vs. nutrients intake in the first week of life, shows that higher lipids **(Ba)** and proteins **(Bb)** intake correlate with less EMA gap. Vertical lines connect individuals’ EEG metrics from T1 and T2; *p*-values refer to GEE models that account for PMA at both recordings. Vertical lines connect individuals’ EEG metrics from T1 and T2; *p*-values refer to GEE models that account for PMA at both recordings.

EMA estimates significantly correlated with the actual age at EEG recording (see Figure 3A). This allowed comparing EMA or its change to the known age (or its change) during each recording session.

A reduced early protein and lipids intake (protein: *B* = 1.108, *p* < 0.001; lipids: *B* = 0.595, *p* = 0.042, *n* = 28) was significantly correlated with a lower EMA. Lower early protein and lipids intake were also significantly associated with a higher EMA gap (proteins: *B* = 1.108, *p* < 0.001; lipids: *B* = 0.595, *p* = 0.042, *n* = 28; see Figures 3Ba,b).

### EEG Maturational Features vs. Clinical Outcome

**Figure 4:**
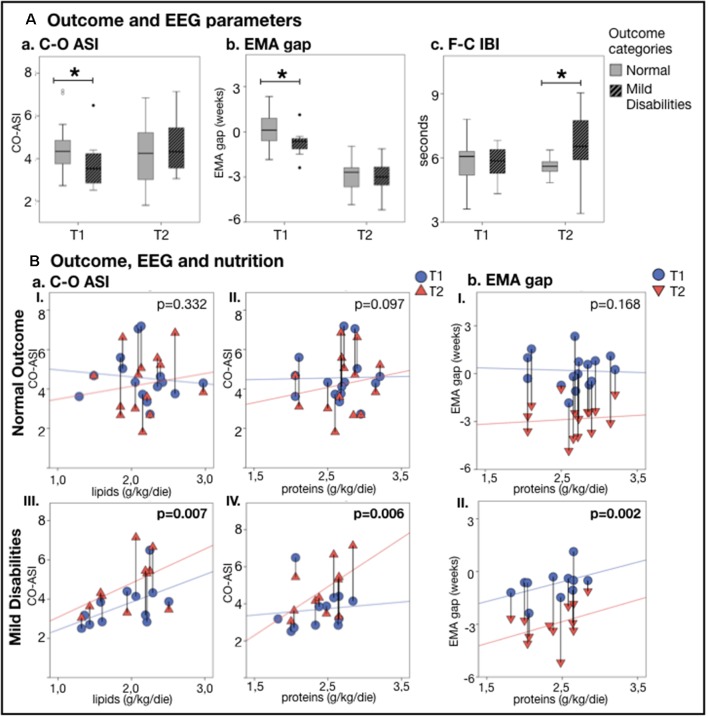
EEG metricsand nutrition vs. neurodevelopmental outcome at 24 months. **(A)** Comparison of outcome groups shows that a typicallydeveloping infant has higher C-O ASI (*p* = 0.045; **Aa**) and lower EMA gap (*p* = 0.030; **Ab**) atthe first recording (T1), while those with minor developmental disabilities had a longer F-C IBI (*p* = 0.009;**Ac**) at the second recording (T2) (*p < 0.05). **(B)** Change in EEG metrics vs. nutrition shows that babieswith mild disabilities presented with a stronger increase in C-O ASI and lower EMA gap as a function of lipids (**aI**typical outcome group, **aIII** atypical outcome group) and proteins (**aII,bI** typical outcome group, **aIV,bII** atypical outcome group). The effect of nutrition on EEG features lessened and lost significance by considering only who developed typically. Vertical lines connect individuals’ EEG metrics from T1 and T2; *p*-values refer to GEE models that account for PMA at both recordings.

The relationships between early EEG measures and clinical outcomes were assessed by comparing infants with typical development (*n* = 16, GA = 27.5 ± 1.8 weeks) and those with minor neurological disabilities (*n* = 12, GA = 29.4 ± 2.0 weeks). First, we examined whether nutritional effects on EEG measures co-vary with the later clinical outcome in the split infant cohorts. The correlation of early protein and lipid intake with Centro-occipital ASI was stronger and significant only in infants with minor disabilities (Figure 4Ba). Infants with disabilities also presented with a stronger relationship between early proteins and the EMA gap (Figure 4Bb). Infants with normal development showed a significant relationship between lipid intake and EMA gap, as well as weaker correlation between caloric intake and developmental changes in global spectra. No group-wise effect was found for MSC.

Second, we studied at the whole group level whether early EEG metrics differed between clinical outcome groups. In the first EEG recording (T1), infants with later development of minor disabilities presented with a higher EMA gap (*p* = 0.030; see Figure 4Ab), lower ASI over the Centro-occipital region (*p* = 0.045; see Figure 4Aa), as well as higher ASI over the fronto-temporal regions (*p* = 0.021). At the second EEG recording (T2), the infants with minor disabilities showed significantly longer IBIs over the fronto-central region (*p* = 0.009; see Figure 4Ac) as well as higher centrotemporal ASI (*p* = 0.033).

## Discussion

Our study showed that nutritional supply in the first weeks after preterm birth influences the maturation of brain activity. Moreover, the present observations suggest that nutritional effects may be different in infants with developmental adversity. Our findings support studies showing EEG abnormalities in preterm infants develop after severely compromised nutrition, a state rarely encountered in contemporary NICUs (Hayakawa et al., 2003; Okumura et al., 2010). Previous MRI work has provided evidence for measurable structural effects of early nutritional balance (Vinall et al., 2013; Beauport et al., 2017; Schneider et al., 2018). Our work extends these structural studies by showing the functional effects of nutritional supply, raising the possibility of developing functional biomarkers for assessing the neurological effects of nutritional stress.

The prevailing view on early neurodevelopmental mechanisms supports the idea that spontaneous cortical activity may be sensitive to the effects of early interventions. The rationale starts from the well-established, intimate structure-function link where rapid structural maturation of brain circuitry is guided and driven by endogenous neuronal activity (Vanhatalo and Kaila, 2006; Hanganu-Opatz, 2010; Luhmann et al., 2016; Keunen et al., 2017; Khazipov and Milh, 2018). There is also an increasing awareness of the sensitivity of early neuronal network activity to a variety of stress factors ranging from metabolic imbalance, such as nutritional stress, medical adversities and medical treatments (Keunen et al., 2015; Leviton et al., 2015; Videman et al., 2016, 2017).

We used parameters that were designed to measure distinct properties, or network mechanisms, to allow some degree of physiologically reasoned interpretation of the results. All findings are compatible with the idea that more effective nutrition, measured by higher nutrient or calorie intake, is generally expediting neurological maturation. Moreover, the effect is seen in many independent EEG parameters, i.e., brain mechanisms: global temporal organization (continuity and spectra), emerging networking activity (interhemispheric synchrony), and a global assessment of functional maturation (EMA index). The recent development of computational algorithms makes these measures available for automatic EEG assessment, opening a venue for early functional biomarkers to benchmark nutritional or other interventions (Koolen et al., 2016; Stevenson et al., 2017).

There are clear limitations in our present work: the sample size was relatively small and based on a retrospective collection of nutritional status. Future work within a prospective nutritional intervention trial would be useful to allow principled assessment of the brain effects of discrete nutritional schemes. The computational EEG measures may be also challenging with respect to their mechanistic interpretability and their potential sensitivity to epoch selection, including remaining artifacts or little variance in parameter settings. Here, we tried to mitigate this concern by using standard visual EEG review in the epoch selection, and the measures used here were generally robust to many artifacts, yet it is essential to validate the findings in independent datasets. We hope to promote such validation work by providing the full algorithms in the open online repositories.

The interpretations of our findings in terms of exact causal mechanisms between nutrition and EEG activity are, however, only speculative. The present dataset was collected as part of a clinical nutrition study, and the EEG measures comprised as an observational sub-section. The retrospective nature of this study and the small number of the recruited subjects, imply therefore a lot of unbalanced variability among their clinical profiles and thus limit inferences of causality between nutrition and brain development. For ethical reasons, many causal mechanisms cannot be studied in human infants where nutritional intervention is defined by the clinical needs determined on a daily basis at the bedside for each infant. Future clinical studies may approach questions of mechanisms statistically when larger cohorts allow more detailed stratifications. For example, we used statistical methods to handle the differences in PMA, which is a potential confounder when the development of EEG measures depends on the infant’s age (O’Toole et al., 2019). We also stratified infants according to neurodevelopment to show that the developmental trajectories and/or nutritional sensitivity are different in these infants at a very early age. While the dataset is too small to allow further analyses, it suggests that infants may have individual developmental trajectories at a very early age, and optimal neurological care could aim to personalize medical treatments according to such EEG phenotyping. The development of such therapeutic strategies will need substantial translational groundwork, which can use EEG metrics as a direct translational bridge across species.

## Conclusion

Our study shows that early nutritional balance after preterm birth may influence subsequent maturation of brain activity in a way that can be observed with several intuitively reasoned and transparent computational EEG metrics. The retrospective nature and the limited size of our present cohort preclude more thorough comparisons of nutritional disparities, however, the findings suggest that the early nutritional imbalance may interfere with brain mechanisms that guide functional wiring in the developing brain. Identification of the cellular level mechanisms is needed to understand the observed effects, and the hereby employed computational EEG metrics may provide translational benchmarks for such experimental works. Moreover, such EEG metrics also hold promise for becoming early functional biomarkers of brain maturation, to be used for benchmarking future therapeutic interventions.

## Data Availability Statement

The datasets generated for this study are available on request to the corresponding author.

## Ethics Statement

The studies involving human participants were reviewed and approved by Azienda Ospedaliera—University Hospital of Pisa. Written informed consent was provided by the participants’ legal guardian/next of kin.

## Author Contributions

VM designed and conducted the study, was responsible for collecting EEG and clinical data, participated in the EEG analysis, drafted and revised the manuscript, and she is also the corresponding author of this publication. PG and SV contributed to the conception and design of the study. FM collected and organized the nutritional database. SS performed the statistical analysis. AG contributed in the collection of clinical assessments, interpretation of results and reviewed and revised the manuscript. SV and NS supervised EEG data collection, contributed to data interpretation, and reviewed and revised the manuscript. NS, NK, and RM performed parts of the EEG analysis. All authors contributed to manuscript revision, read and approved the submitted version.

## Conflict of Interest

The authors declare that the research was conducted in the absence of any commercial or financial relationships that could be construed as a potential conflict of interest.
